# Factors Influencing Dislodgeable 2, 4-D Plant Residues from Hybrid Bermudagrass (*Cynodon dactylon* L. x *C*. *transvaalensis*) Athletic Fields

**DOI:** 10.1371/journal.pone.0148992

**Published:** 2016-02-10

**Authors:** Matthew D. Jeffries, Travis W. Gannon, James T. Brosnan, Khalied A. Ahmed, Gregory K. Breeden

**Affiliations:** 1 Department of Crop Science, North Carolina State University, Raleigh, North Carolina, United States of America; 2 Department of Plant Sciences, University of Tennessee, Knoxville, Tennessee, United States of America; Ghent University, BELGIUM

## Abstract

Research to date has confirmed 2,4-D residues may dislodge from turfgrass; however, experiments have not been conducted on hybrid bermudagrass (*Cynodon dactylon* L. x *C*. *transvaalensis*), the most common athletic field turfgrass in subtropical climates. More specifically, previous research has not investigated the effect of post-application irrigation on dislodgeable 2,4-D residues from hybrid bermudagrass and across turfgrass species, research has been nondescript regarding sample time within a d (TWD) or conducted in the afternoon when the turfgrass canopy is dry, possibly underestimating potential for dislodgement. The effect of irrigation and TWD on 2,4-D dislodgeability was investigated. Dislodgeable 2,4-D amine was reduced > 300% following irrigation. From 2 to 7 d after treatment (DAT), ≤ 0.5% of applied 2,4-D was dislodged from irrigated turfgrass, while ≤ 2.3% of applied 2,4-D was dislodged when not irrigated. 2,4-D dislodgeability decreased as TWD increased. Dislodgeable 2,4-D residues declined to < 0.1% of the applied at 1 DAT– 13:00, and increased to 1 to 3% of the applied 2 DAT– 5:00, suggesting 2,4-D re-suspended on treated turfgrass vegetation overnight. In conclusion, irrigating treated turfgrass reduced dislodgeable 2,4-D. 2,4-D dislodgeability increased as TWD decreased, which was attributed to non-precipitation climatic conditions favoring turfgrass canopy wetness. This research will improve turfgrass management practices and research designed to minimize human 2,4-D exposure.

## Introduction

Turfgrasses are grown on over 16.3 million hectares in the contiguous United States (US)–exceeding the combined area of irrigated grain corn [(*Zea mays* L.) 2.5 million], soybeans [*Glycine max* L.) 2.1 million] and cotton [(*Gossypium hirsutum* L.) 0.9 million]–and are utilized by the public with land uses including commercial/residential lawns, golf courses, parks and roadsides [1, 2]. Turfgrasses on athletic grounds and facilities are also widespread, with > 700,000 managed athletic fields in 2003 [3]. The US Census Bureau reported in 2009 over 40% of the population ages 7 to 44 participated in baseball, football, golf, soccer and/or softball, all played predominately on managed natural turfgrass [4]. Providing an acceptable playing surface poses many challenges for athletic field managers. The surface must be aesthetically pleasing, functional and safe to the end-user. Public athletic fields are often overused or used when environmental conditions favor playing surface degradation. Consequently, weed encroachment may occur that adversely affects surface strength and uniformity. Ultimately, player safety may be compromised due to poor footing conditions manifesting as playing surfaces degrade [5, 6]. To mitigate these issues, synthetic herbicides are commonly applied for weed control on athletic fields.

Major pathways of direct human exposure to pesticides in turfgrass systems include inhalation (dust/vapor), nondietary ingestion and dermal contact [7]. 2,4-dimethylamine salt (2,4-D) is a selective postemergence broadleaf herbicide currently registered for use in numerous US crops and non-cropland areas. In 2005, over 650 products containing 2,4-D were registered in over 300 distinct agricultural and residential use sites [8]. At that time, it was reported 7.3 million kg 2,4-D (34% of total US use) were applied to non-cropland areas including athletic fields [8]. While 2,4-D is routinely applied in numerous commodities worldwide, its use has been questioned since the 1970’s due to toxicological concerns [9, 10]. Research regarding 2,4-D carcinogenicity, as well as its effect on neurologic and reproductive processes is inconclusive; however, it is a known toxin to blood (reduction in hemoglobin and red blood cells), the liver (decreased enzyme activity) and the kidney (increased organ weight) [8, 11, 12]. Furthermore, acute 2,4-D exposure is an eye irritant and chronic 2,4-D oral exposure experiments on animals have resulted in damage to the eye, thyroid, kidney, adrenals and ovaries/testes [8].

Following a pesticide application to turfgrass, numerous transfer and transport processes ensue. Chemical properties pertaining to 2,4-D transport from the intended site include: very high water solubility (K_s_ = 796,000 mg L^-1^; 20°C), short soil half-life (T_1/2_ = 6.2 d), low volatility (vapor pressure = 1.0 x 10^−7^ mm Hg) and moderate soil organic carbon sorption coefficient (K_oc_ = 20 mL g^-1^) [8,13,14]. These properties suggest 2,4-D may readily dislodge from treated turfgrass vegetation onto humans, which has been confirmed most notably by Nishioka et al. [15] and Morgan et al. [16]. In these independent studies, samples were collected from various areas within homes prior to, and following 2,4-D applications to the residential lawns. The authors detected 2,4-D in 122 of the 142 homes sampled (across two states and three populations) [15, 16]. These data confirm 2,4-D transfers from turfgrass vegetation to off-target areas since it was not applied inside, nor is labeled for indoor use. If transferred from treated turfgrass vegetation, 2,4-D human non-occupational absorption occurs via dietary and non-dietary ingestion, and to lesser extent through skin [15–18]. Once 2,4-D is absorbed into the body, it is not metabolized and is rapidly eliminated in urine [16]. For this reason, human 2,4-D exposure is typically measured via urine samples, and has been commonly detected in children’s urine, confirming exposure [16, 19, 20].

Unlike most traditional agricultural settings, non-occupational re-entry into areas recently treated with pesticides is common and lawful in most turfgrass systems throughout many regions of the US. In most states, a specific non-occupational re-entry interval is not required following a pesticide application to athletic field turfgrass. With many pesticides, as long as the product has dried, re-entry is permissible. Plant canopies in established turfgrass systems inherently intercept sprayed pesticides. Pesticide in/on turfgrass vegetation is subject to dislodge onto maintenance equipment or players, increasing human pesticide exposure potential [21]. Typically, due to reported short half-lives (< 7 d) in turfgrass vegetation, pesticide dislodgement off of the treated area is not considered a long-term concern; however, appreciable amounts can be dislodged in the h and d immediately following an application [22, 23]. Sears et al. [24] reported 10% of the applied diazinon was dislodged by wiping cheesecloth over the treated area immediately after application with ten strokes in opposing directions. Similarly, Harris and Solomon [25] conducted an experiment simulating a recreational setting and reported 8% of the applied 2,4-D was dislodged onto running shoes 1 h following application.

Previous research indicates dislodgeable pesticide residues on plants may be reduced by applying granular products in lieu of spray applications, as well as by irrigating treated areas following an application [21, 26, 27]. Thompson et al. [28] reported liquid applied 2,4-D was up to 15 times more dislodgeable than granular 2,4-D following an application. The authors also reported < 0.01% of the applied 2,4-D liquid formulation was dislodgeable after 1 d when a rainfall event (18 mm) occurred 1 h after treatment, suggesting post-application irrigation may reduce dislodgeable 2,4-D plant residues [28]. While this information is valuable for reducing dislodgeable 2,4-D plant residues, it should be noted granular applied 2,4-D and irrigation/rainfall 1 h after spraying 2,4-D may compromise weed control [29, 30]. Additional research is needed to evaluate dislodgeable 2,4-D plant residues when applied as a liquid formulation and irrigation is delivered at an agronomically sound timing.

To our knowledge, research to date has not evaluated dislodgeable 2,4-D plant residues on hybrid bermudagrass athletic fields. Hybrid bermudagrass is the most commonly managed turfgrass species on athletic fields in tropical and subtropical (latitudes ≈ 45°N to 45°S) regions of the world due to its tolerance to low mowing heights (≥ 0.6 cm) coupled with its comparatively superior recuperative abilities following periods of heavy traffic [31]. Currently, published 2,4-D dislodgement research has predominantly been conducted on Kentucky bluegrass (*Poa pratensis* L.), a cool-season turfgrass species that possesses different growth characteristics than bermudagrass [31]. Specifically, Kentucky bluegrass has an erect, bunch-type growth habit and a C_3_ photosynthetic pathway. The presented research was conducted on a prostrate growing (rhizomes/stolons), C_4_ turfgrass. In addition, previous research has shown turfgrass species with similar growth characteristics commonly differ with regards to herbicide uptake, translocation and metabolism, which further supports the need for turfgrass species-specific research on dislodgeable pesticide residues [32–34].

Research has not been published quantifying pesticide dislodgeability from turfgrass over time within a d (TWD). In many regions, turfgrass canopy dynamics, including surface moisture, fluctuate throughout the day. As moisture increases, compounds may re-suspend on turfgrass vegetation, and therefore may be more dislodgeable if not tightly bound to vegetation (i.e. high water solubility and low to moderate binding affinity). Finally, published research to date has not encompassed 2,4-D dislodgeability in a setting simulating the most popular international sport, soccer [35]. Beyond its popularity, research evaluating 2,4-D dislodgeability is warranted due to the inherent frequency of ball-to-turfgrass contact that occurs in this sport. If 2,4-D dislodges from treated turfgrass onto the ball human exposure may occur via numerous routes, most notably by hand contact during certain procedures within games/practices.

The objectives of this research were to quantify dislodgeable 2,4-D plant residues over time on a hybrid bermudagrass athletic field surface, as well as to elucidate the effect of irrigation and natural surface moisture on 2,4-D dislodgeability from hybrid bermudagrass turfgrass. We hypothesized irrigation and conditions favoring turfgrass canopy dryness would reduce 2,4-D dislodgeability on athletic fields.

## Materials and Methods

### Research Overview

Two field experiments were conducted and repeated in time in North Carolina to evaluate dislodgeable 2,4-D plant residues from hybrid bermudagrass. Experiment 1 was designed to quantify the effect of irrigation on dislodgeable 2,4-D plant residues. 2,4-D-treated plots were either irrigated (0.3 cm H_2_O) or not irrigated 24 h following treatment. Samples characterizing 2,4-D dislodgement from turfgrass were collected from 7:00 to 9:00 EST at various d after treatment (DAT). Results from this experiment suggested dislodgeable 2,4-D residues declined over time within a sampling date, which we hypothesized was due to a reduction in turfgrass canopy moisture as dew/distillation/guttation fluid dissipated off vegetation. To investigate this observation, an additional experiment was developed. Experiment 2 was designed to quantify the effect of TWD on dislodgeable 2,4-D plant residues. In short, 2,4-D treated plots were sampled every 2 h from 5:00 to 13:00 EST at various DAT to elucidate the effect of turfgrass canopy surface moisture on dislodgeable 2,4-D residues.

### Site Description

Experiment 1 was initiated June 10, 2013 and May 28, 2014 (Thomas E. Brooks Park, Cary, NC, USA; Lat. 35°47’41.74” N, Long. 78°53’50.37” W) on a sand textured soil with pH 6.9 and 1.5% organic matter (OM) w w^-1^. Experiment 2 was initiated August 27, 2013 and August 26, 2014 (Lake Wheeler Turfgrass Field Lab, Raleigh, NC, USA; Lat. 35°44’21.34” N, Long. 78°40’49.75” W) on a sandy clay loam soil with pH 6.4 and 1.9% OM w w^-1^.

Both experiments were conducted on weed-free, established hybrid bermudagrass [*Cynodon dactylon* (L.) Pers. × *Cynodon transvaalensis* Burtt-Davey, cv. ‘Tifway 419’] areas maintained at a 3 cm height of cut where 2,4-D had not been applied 2 yr preceding initiation. Prior to experiment initiation, vegetation and soil samples from the areas were analyzed to confirm 2,4-D residues were non-detectable. Select climatic conditions were logged throughout experiments. Finally, leaf wetness (Leaf Wetness Sensor; Decagon Devices Inc., Pullman, WA, USA) was measured throughout experiment 2 with a flat-plate sensor placed facing north at a 45° angle from the ground surface and 0.6 m height.

### Experimental Design

Experiment 1 was conducted as a split plot, randomized complete block design with three replicates of a 2-by-7 factorial treatment arrangement. Main plots were split by irrigation (irrigated or non-irrigated) with seven subplot sample timings (1, 2, 5, 7, 14, 21 or 28 DAT). Experiment 2 was conducted as a randomized complete block design with three replicates of a 5-by-5 factorial treatment arrangement. Factors included sample collections at five TWD (5:00, 7:00, 9:00, 11:00 or 13:00 EST) in each of five DAT (1, 2, 3, 6 or 12 DAT). Samples were also collected for both experiments 1 h after application and immediately following application for experiment 2; however, these samples were not included in statistical analyses due to differing collection timings from other samples beyond 0 DAT. A nontreated check was included in all experimental blocks to ensure the trial area was not contaminated.

### Experiment Initiation

One d prior to trial initiation, areas were mown (clippings collected) and irrigated to field capacity. Experimental areas were not irrigated or mown for 7 d following treatment. Furthermore, areas were covered (HDX 6 Mil Clear Plastic; The Home Depot Corp., Atlanta, GA, USA) during rainfall events during this 7 d period. At experiment initiation, 2,4-D amine (Amine 400 2,4-D Weed Killer^®^; PBI/Gordon Corp., Kansas City, MO, USA) was applied at 2.1 kg ai ha^-1^ to plots measuring 1.5 by 2.25 m (1 m alleys between reps). Treatments were sprayed at 14:00 to allow the solution to dry on the vegetation during ≥ 5 h of sunlight. Applications were made with a hand-held CO_2_-pressurized sprayer comprised of four 80015 XR VS flat-fan nozzles (TeeJet^®^ Flat-Fan Nozzles; Spraying Systems Co., Wheaton, IL, USA). The carrier volume selected (187 L ha^-1^ at 179 kPa) is the minimum stated on the label, creating the worst-case scenario for pesticide retention on the turfgrass canopy. Also, it should be noted that the 2,4-D application rate used in this research was 20% higher than currently allowed on athletic fields. This was done based on preliminary testing that confirmed an increased rate was necessary to ensure 2,4-D residue detection up to 7 DAT, which we felt was a justifiable compromise to better elucidate the effects of the research variables of interest. Finally, to ensure 2,4-D was applied at the intended rate over the trial area, water-filled glass containers (15.5 cm^2^; 65 mL HPLC-grade H_2_O) were randomly placed throughout the trial area. Following 2,4-D application overtop the containers, the residue concentration in these containers was quantified by high performance liquid chromatography (HPLC) with a diode array detector (DAD) analysis.

Irrigation treatments for experiment 1 were applied at 1 DAT (13:00 EST) with an eight-nozzle boom equipped with 8008 XR VS flat-fan nozzles that was calibrated at 172 kPa to deliver 0.3 cm H_2_O plot^-1^ with four passes. This approach to simulate irrigation was conducted due to logistical considerations for the research area and experimental design, as well as to ensure uniform irrigation amount/intensity and minimize variability in the data. Furthermore, the output rate and the droplet size produced by the nozzle-pressure combination of our boom both fall within the spectrum delivered by various commercially available impact and rotary style irrigation heads in turfgrass systems [36–38].

### Sample Collection

#### Total 2,4-D in/on Turfgrass Vegetation

Total 2,4-D in/on turfgrass vegetation was quantified at all sample collection timings to: 1) use as a reference point for the total amount of 2,4-D dislodged from hybrid bermudagrass over time (section 2.7, Eq 1); and 2) characterize 2,4-D dissipation in/on hybrid bermudagrass over time. This was done by collecting a core (10.8 cm diam; 92 cm^2^) such that sampling equipment did not contact aboveground vegetation. Following collection, all samples were frozen, aboveground vegetation was harvested, weighed, processed [1.7 mm (Fitzmill Homoloid Model JT 6; Fitzpatrick Co., Elmhurst, IL, USA)] and stored at -12°C until extraction and residue analysis.

#### Total Dislodgeable 2,4-D

Dislodgeable 2,4-D was quantified by rolling a soccer ball (Franklin Sports Competition 100 Soccer Ball, Size 4; Franklin Sports, Stoughton, MA, USA) over a 9 m distance (four 2.25 m side-by-side rolls) within a unique plot. Ball roll distance was based off of a soccer ball rolling 50% of the recommended field length (18 m) for youth ages < 6 yr in the US [39]. The soccer ball was double-wrapped with a 5 by 120 cm absorbent strip (100% Cotton Cheesecloth; Chef Revival, North Charleston, SC, USA) selected by previous researchers investigating related processes [24, 25, 28]. The soccer ball was mounted to a hand-held PVC apparatus designed such that the ball rotated end over end in the same direction as the absorbent strip, thus allowing for constant absorbent strip contact to the treated turfgrass surface. While this is not representative of a ball roll when actively playing soccer, this measure was required to minimize variation in data and determine the maximum dislodgeable 2,4-D from turfgrass vegetation. Following ball roll, the absorbent strip was removed, placed in a unique glass jar (473 cm^3^) and stored at -12°C for subsequent extraction and HPLC-DAD analysis.

### Residue Analyses

#### Total 2,4-D in/on Turfgrass Vegetation

All reagents and solvents used for residue analyses were HPLC grade. Total 2,4-D residue in/on turfgrass vegetation was quantified with modifications to methods by Shin et al. [40]. Processed vegetation (5 g) was mixed with water (100–120 mL) in a high-speed homogenizer [9000 rpm (VIRTIS 45 Homogenizer; The VIRTIS Co., Gardiner, NY, USA)] for 2 min. After mixing, samples settled (10 min) and an aliquot (10 mL) was centrifuged [3500 rpm (Allegra 6-KR Centrifuge; Beckman Coulter Inc., Brea, CA, USA)] for 10 min. A sub-aliquot (7 mL) was then taken and sodium chloride (1 g) was added and partitioned with n-hexane (4 mL). The upper hexane layer was discarded and this partition was repeated once. The aqueous solution was acidified with 10% sulfuric acid (0.5 mL) to reach pH < 2. The acidified aqueous solution was partitioned twice with dichloromethane [6 mL total (DCM)] and evaporated to dryness using N-evap (N-EVAP 112; Organomation Associates Inc., Berlin, MA, USA). Samples were reconstituted in water + acetonitrile [7 mL (9 + 1 by volume)]. Samples were sonicated for 3 min (Branson 2510 Ultrasonic Cleaner; Branson Ultrasonic Co., Danbury, CT, USA) and vortexed for 3 min. Samples were then vialed for injection.

#### Total Dislodgeable 2,4-D

Total dislodgeable 2,4-D residues were quantified with modifications to methods by Snyder and Cisar. [41]. Prior to extraction, samples were allowed to thaw at room temperature (22°C) for 30 min. Water (100 mL) was added to each sample and shaken [300 rpm (KS 501 Digital Shaker; IKA Works, Inc., Wilmington, NC, USA)] for 1 h. After shaking, extract was decanted, the absorbent strip was compressed to remove additional solution and the extraction process was repeated with water (25 mL). Extracts were combined and mixed for 5 min and an aliquot (50 mL) was taken and centrifuged (3500 rpm) for 10 min. Each centrifuged sample (1 mL) was filtered (0.45 μm nylon filter; Thermo Fisher Scientific, Inc., Pittsburgh, PA, USA) and vialed for injection.

#### Analytical Parameters

2,4-D residues were quantified for cheesecloth, vegetation and water matrices by HPLC-DAD (Agilent-1260 Infinity; Agilent Technologies, Inc., Wilmington, DE, USA). High performance liquid chromatography parameters included: C_18_ silica column [75 mm length by 4.6 mm i.d. (Poroshell 120 EC-C18; Agilent Technologies, Inc., Wilmington, DE, USA)]; 32°C column temperature; acetonitrile + water (3 + 2 by volume) + 0.1% phosphoric acid by volume mobile phase; 1 mL min^-1^ flow rate; 10 μL injection volume. 2,4-D retention time was 0.6 min at 230 nm. Limits of detection and quantification were 0.3 and 1.0 mg L^-1^ respectively, while maintaining the signal to noise ratio at 3:1. Pesticide concentrations were quantified using peak area measurements (OpenLAB CDS ChemStation, Version C.01.04; Agilent Technologies, Inc., Wilmington, DE, USA). Residue concentrations above the calibration curve were diluted and re-injected for analysis. Finally, fortification recovery checks for cheesecloth, vegetation and water matrices ranged from 90 to 101, 85 to 97 and 98 to 102%, respectively, across all analyses conducted in the presented research.

#### Dislodgement Calculations

From the data collected, 2,4-D dislodgeability is presented in two ways. The first is only reported for experiment 1, which is dislodgement relative to the total amount in/on turfgrass vegetation at a given point in time that was calculated using the equation:

$$\%\text{ dislodgment }=\;\left[\left(\text{BR }\mu g{\text{ 2,4-D cm}}^{-2}/\text{ AV }\mu g{\text{ 2,4-D cm}}^{-2}\right)\;x\;100\right]$$ (1)

where BR and AV represent 2,4-D residues recovered from ball roll and turfgrass vegetation samples, respectively. The second reporting method was completed for ball roll and turfgrass vegetation recoveries in both experiments, which was 2,4-D dislodgement relative to the amount applied at trial initiation using the equation:

% dislodgment = [(BR μg 2,4-D cm−2/ 20.9 μg 2,4-D cm−2) x 100] (2)

where BR represents 2,4-D residue recovered from ball roll samples relative to the 2,4-D application rate (20.9 μg 2,4-D cm^-2^).

#### Statistical Analysis

Statistical analyses were conducted by analysis of variance (P = 0.05) using MIXED procedures in SAS (Statistical Analysis Software^®^, Version 9.2; SAS Institute, Inc., Cary, NC, USA). Irrigation and TWD were considered fixed effects for experiments 1 and 2, respectively, while DAT was considered a fixed effect for both experiments. Main effects and their interactions are presented accordingly, with precedent given to significant interactions of increasing magnitude [42]. Means were separated according to Fisher’s protected LSD (P < 0.05) and Pearson correlation coefficients (P = 0.05) were determined to quantify the relationships between selected climatic conditions and leaf wetness with dislodgeable 2,4-D plant residues.

## Results

### Application Recovery Checks and Experimental Runs

Application recovery check containers determined 2,4-D was applied at 93 to 106% of the intended rate for all experiments and experimental runs. In general, data trends between runs of experiment 1 were similar; however, dislodgeable 2,4-D residues were detected for a longer period of time following application in run 2. The authors attribute this to improved methods for covering plots during periods of rainfall in run 2. Plots were covered in run 1 of both experiments by securing plastic to the ground during rainfall events. While turfgrass vegetation was always dry when plots were covered, ground distillation coupled with evapotranspiration caused the accumulation of water droplets on the underside of the plastic. Although measures were taken to minimize this occurrence and prevent contamination across plots, there was likely an effect on dislodgeable 2,4-D residues via losses sorbed to plastic or soil/thatch. To address this concern, PVC structures were constructed prior to run 2 of both experiments to prevent plastic contact with treated turfgrass vegetation during rainfall events. Consequently, experimental runs were analyzed and are presented separately for experiment 1, while experimental runs were pooled for experiment 2.

### Effect of Irrigation on 2,4-D Dislodgeability

#### Dislodge of Total in/on Turfgrass Vegetation

2,4-D residues were not detected on turfgrass vegetation beyond 7 DAT; therefore, data from 14 to 28 DAT were excluded from statistical analyses. Overall, 2,4-D dislodgement as a percent of the total in/on turfgrass vegetation at a given point in time was negligibly affected by irrigation or DAT (Table 1). In run 1 an irrigation-by-DAT interaction was detected; however, no differences were detected between irrigation treatments at 2 DAT, which is the first sampling following irrigation. However, in run 2 a greater proportion of the total 2,4-D in/on turfgrass vegetation was dislodged from non-irrigated plots at 2 and 5 DAT. Across runs, no differences were detected at 7 DAT.

**Table 1:** The effect of irrigation on dislodgeable 2,4-D residues relative to the total in/on turfgrass vegetation.^a–c^

	^______________^ Run 1 ^____________^		^____________^ Run 2 ^______________^	
DAT	Irrigated	Non-irrigated	Irrigated	Non-irrigated
	^________^ % dislodged of total in/on turfgrass vegetation ^________^			
1	2.3	2.3	5.3	3.9
2	2.8	2.2	1.2	4.2
5	< LOD	2.1	3.5	5.7
7	< LOD	0.1	4.9	5.1
14	< LOD	< LOD	< LOD	< LOD
21	< LOD	< LOD	< LOD	< LOD
28	< LOD	< LOD	< LOD	< LOD
LSD_0.05_^d^	^_______________^ 0.8 ^_______________^		^_______________^ 1.7 ^_______________^	

^a^ Abbreviations: DAT, d after treatment (2,4-D); LOD, limit of detection; NS, not significant.

^b^ 0.3 cm H_2_O irrigation applied at 1 DAT (13:00 EST) after sampling.

^c^ Data from 14 to 28 DAT not included in statistical analysis.

^d^ LSD (P < 0.05) for comparing irrigation and DAT within run.

While minimal differences in dislodgment were detected as a percentage of the total load of 2,4-D in/on turfgrass vegetation, the quantity of 2,4-D in/on turfgrass vegetation was affected by irrigation and DAT in both runs (Table 2). In neither experimental run were differences detected prior to irrigation (i.e., 1 DAT sampling); however, non-irrigated plots retained more 2,4-D at 2 and 5 DAT compared to irrigated vegetation. In run 1, 2,4-D retention was reduced 72% at 2 DAT by irrigating plots. Furthermore, < 7% of applied 2,4-D remained in/on turfgrass vegetation at 5 and 7 DAT when irrigation was applied. Although less pronounced in run 2, all irrigated samples collected from 2 to 7 DAT retained less 2,4-D than non-irrigated vegetation. Finally, 2,4-D was not detected in/on turfgrass vegetation from 14 DAT until the end of the study in both experimental runs.

**Table 2:** The effect of irrigation on 2,4-D in/on turfgrass vegetation.^a–c^

	^_____________^ Run 1 ^____________^		^____________^ Run 2 ^_____________^	
DAT	Irrigated	Non-irrigated	Irrigated	Non-irrigated
	^____________________________^ % of applied ^____________________________^			
1	82	93	71	73
2	21	74	34	57
5	6	19	26	40
7	2	11	17	37
14	< LOD	< LOD	< LOD	< LOD
21	< LOD	< LOD	< LOD	< LOD
28	< LOD	< LOD	< LOD	< LOD
LSD_0.05_^d^	^_______________^ 14 ^_______________^		^_______________^ 8 ^_______________^	

^a^ Abbreviations: DAT, d after treatment (2,4-D); LOD, limit of detection.

^b^ 0.3 cm H_2_O irrigation applied at 1 DAT (13:00 EST) after sampling.

^c^ Data from 14 to 28 DAT not included in statistical analysis.

^d^ LSD (P < 0.05) for comparing irrigation and DAT within run.

#### Dislodge of Applied

In both experimental runs, dislodgeable 2,4-D residues (relative to the applied) were reduced > 58% following irrigation (Table 3). At 2 DAT and beyond, ≤ 0.5% of applied 2,4-D was dislodged from irrigated vegetation. On non-irrigated plots, > 1.5% of applied 2,4-D was dislodged 2 DAT (in both experimental runs), as well as 5 and 7 DAT in run 2. Finally, data from this research suggested 2,4-D dislodgeability fluctuated after application. At 0 DAT– 13:00 (1 h following treatment when vegetation had dried) 0.3% of the applied was dislodged (data not shown), which increased to 2.1% the following morning. From this observation the effect of TWD was further elucidated.

**Table 3:** The effect of irrigation on dislodgeable 2,4-D residues relative to the applied at trial initiation.^a–c^

	^_____________^ Run 1 ^____________^		^____________^ Run 2 ^_____________^	
DAT	Irrigated	Non-irrigated	Irrigated	Non-irrigated
	^______________________^ % dislodged of applied ^______________________^			
1	1.8	2.1	3.2	2.7
2	0.5	1.6	0.4	1.7
5	< LOD	0.4	0.9	2.3
7	< LOD	0.0	0.8	1.9
14	< LOD	< LOD	< LOD	< LOD
21	< LOD	< LOD	< LOD	< LOD
28	< LOD	< LOD	< LOD	< LOD
LSD_0.05_^d^	^_______________^ 0.5 ^_______________^		^_______________^ 1.1 ^_______________^	

^a^ Abbreviations: DAT, d after treatment (2,4-D); LOD, limit of detection.

^b^ 0.3 cm H_2_O irrigation applied at 1 DAT (13:00 EST) after sampling.

^c^ Data from 14 to 28 DAT not included in statistical analysis.

^d^ LSD (P < 0.05) for comparing irrigation and DAT within run.

### Effect of Time Within a Day on 2,4-D Dislodgeability

#### Dislodge of Applied

**Fig 1:**
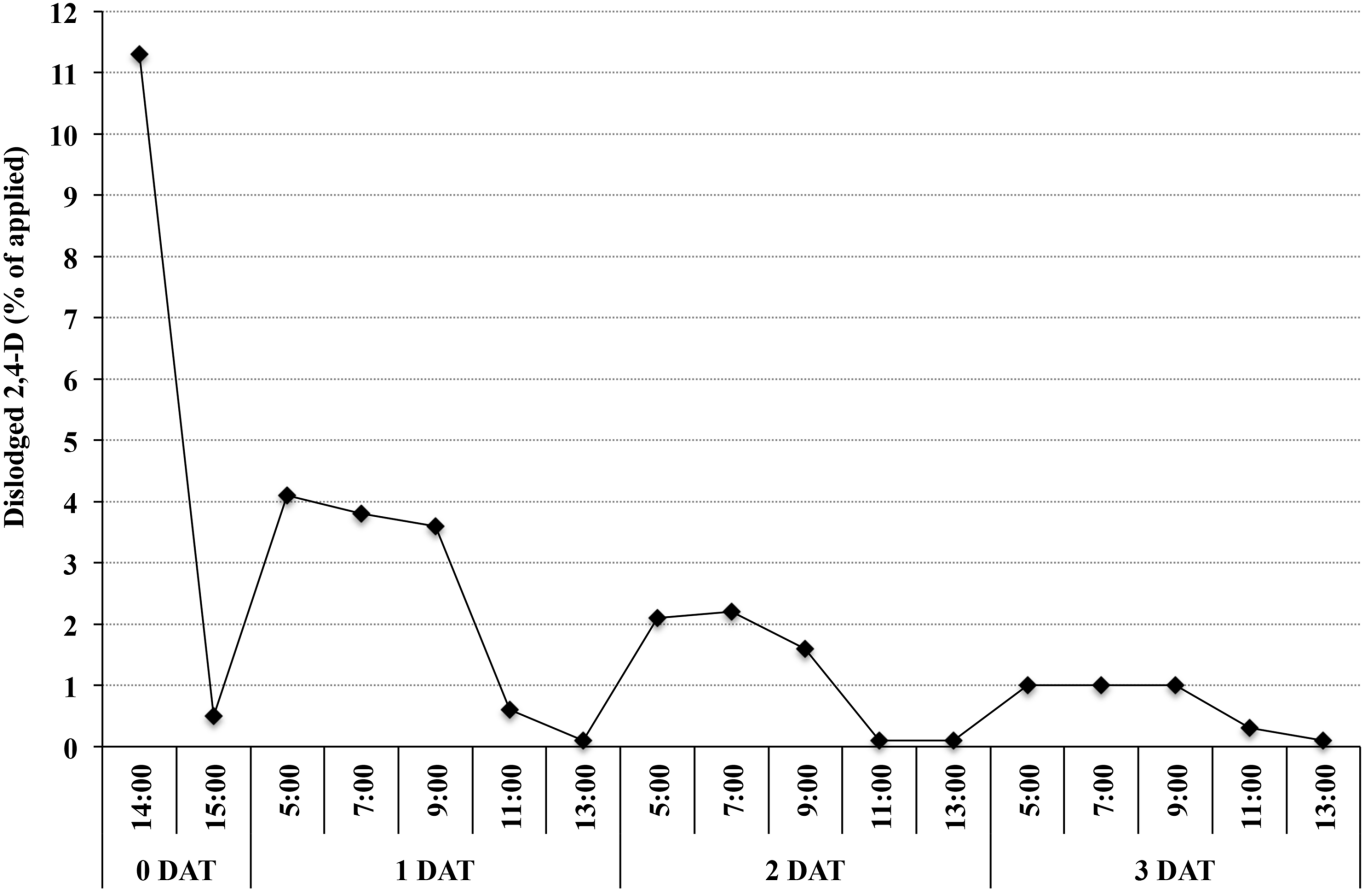
Dislodgeable 2,4-D residues fluctuate over time. Percent dislodged following one soccer ball roll (3.7 m) from hybrid bermudagrass of the applied (2.1 kg 2,4-D ha^-1^) at 14:00 (immediate) and 15:00 on 0 d after treatment (DAT), and from 5:00 to 13:00, 1 to 3 DAT. All sample collection times were eastern standard time.

2,4-D residues were not detected on turfgrass vegetation beyond 6 DAT; therefore, data from 12 DAT were excluded from statistical analyses. 2,4-D residues were not detected beyond 3 and 6 DAT in run 1 and 2 respectively; therefore, data beyond these times were excluded from statistical analyses. A significant DAT-by-TWD interaction was detected (Table 4). In general, 2,4-D dislodgeability decreased as TWD increased (within a DAT), and decreased as DAT increased (within a TWD). Maximum dislodgement was observed at 1 DAT– 5:00, 7:00 and 9:00, with 3.6 to 4.0% of applied 2,4-D dislodged. At these three TWD timings, 2,4-D dislodgement decreased from 1 to 6 DAT; however, no differences were detected between timings within a DAT. With the exception of two sample timings (1 and 3 DAT– 11:00), ≤ 0.1% of applied 2,4-D was dislodged at 11:00 and 13:00 from 1 to 6 DAT. When comparing TWD across DAT in both experimental runs, data suggested 2,4-D re-suspended on turfgrass vegetation overnight, as 2,4-D dislodgement at 1 DAT– 13:00 (0.1% of applied 2,4-D) was less than 2 DAT– 5:00 (2.1%; Fig 1). While not statistically significant, there was also a ten-fold increase in 2,4-D dislodged from 2 DAT– 13:00 (0.1% of applied 2,4-D) to 3 DAT– 5:00 (1%).

**Table 4:** The effect of TWD following application on dislodgeable 2,4-D residues relative to the applied at trial initiation.^a, b^

TWD^c^	1 DAT	2 DAT	3 DAT	6 DAT	12 DAT
	^____________________________^ % dislodged of applied ^____________________________^				
5:00	4.0	2.1	1.0	0.2	< LOD
7:00	3.8	2.2	1.0	0.2	< LOD
9:00	3.6	1.6	1.0	0.1	< LOD
11:00	0.6	0.1	0.3	< LOD	< LOD
13:00	0.1	0.1	0.1	< LOD	< LOD
LSD_0.05_^d^	^_______________________________^ 1.5 ^_______________________________^				

^a^ Abbreviations: TWD, time within a d; DAT, d after treatment.

^b^ Data from 12 DAT not included in statistical analysis.

^c^ Eastern standard time.

^d^ LSD (P < 0.05) for comparing DAT by TWD interaction.

#### Climatic Condition Correlations with Dislodgeability

A review of climatic conditions from 0 to 6 DAT suggested 2,4-D dislodgement may be influenced by climatic conditions that affect turfgrass canopy moisture (Table 5). Relative humidity (RH) is a dimensionless ratio, expressed as a percent of the amount of atmospheric moisture present relative to saturated air [43]. Dew point (DP) is the air temperature (AT) below which moisture in the air condenses, forming dew [44]. Baier [45] and Wilson et al. [46] reported peak dew formation occurred at, or just beyond, sunrise. Hughes and Brimblecombe [47] reported dew formation on velvetgrass (*Holcus lanatus* L.) over a 7 mo period was never observed without guttation, or the emergence of liquid within plants via hydathodes along leaf margins. While these parameters do not solely influence turfgrass canopy moisture, increasing RH, decreasing differences between AT and DP and decreasing time from sunrise (TFS) suggest conditions become more favorable for turfgrass canopy moisture development. Following canopy moisture development, 2,4-D may more readily dislodge from vegetation into solution on treated turfgrass surfaces, and residues may be more readily transferred onto soccer balls. From 1 to 3 DAT, RH was positively correlated with dislodgeable 2,4-D residues (*r =* 0.57 to 0.69; P ≤ 0.01), while negative correlations were observed between dislodgeable 2,4-D and the difference between AT and DP (*r* = -0.55 to -0.73; P ≤ 0.01) as well as TFS (*r* = -0.58 to -0.82; P ≤ 0.001) (Table 6). Correlations weakened as DAT increased, with decreases in all aforementioned comparisons to < 0.5 (+/-) at 6 DAT. Finally, 2,4-D dislodgeability was poorly to moderately positively correlated with leaf wetness from 1 to 6 DAT (*r* = 0.23 to 0.58).

**Table 5:** Climatic conditions recorded for experiment 2 from 1 to 6 DAT.^a, b^

		_______________________ 2013 _______________________				_______________________ 2014 _______________________			
		RH	AT—DP	TFL	LW	RH	AT—DP	TFL	LW
DAT	TWD^c^	(%)	(°C)	(min)	(counts)	(%)	(°C)	(min)	(counts)
1	5:00	91	1.5	-104	207	87	2.2	-103	207
	7:00	91	1.6	16	155	82	3.1	17	111
	9:00	71	5.7	136	3	63	7.4	137	2
	11:00	59	8.7	256	1	53	10.1	257	0
	13:00	61	8.2	376	2	49	11.6	377	0
2	5:00	83	3.1	-104	6	84	2.6	-103	157
	7:00	76	4.4	16	4	79	3.7	17	13
	9:00	71	5.7	136	2	68	6.4	137	3
	11:00	70	5.8	256	1	57	9.3	257	0
	13:00	61	8.1	376	0	50	11.5	377	0
3	5:00	90	1.6	-105	198	90	1.7	-104	9
	7:00	91	1.6	15	176	90	1.7	16	11
	9:00	78	3.9	135	78	80	3.7	136	4
	11:00	59	8.3	255	0	72	5.3	256	2
	13:00	47	12.3	375	0	65	7.1	376	2
6	5:00	91	1.5	-107	98	91	1.5	-106	78
	7:00	92	1.4	13	52	91	1.5	14	101
	9:00	82	3.3	133	6	73	5.1	134	4
	11:00	72	5.4	253	2	58	9.2	254	1
	13:00	64	7.4	373	0	42	14.3	274	0

^a^ Abbreviations: DAT, d after treatment; TWD, time within a day; RH, relative humidity; AT, air temperature; DP, dew point; TFS, time from sunrise; LW, leaf wetness.

^b^ Climatic conditions recorded on site at the Lake Wheeler Turfgrass Field Lab (Raleigh, NC, USA).

^c^ Eastern standard time.

**Table 6:** Pearson correlation coefficients for experiment 2 quantifying the relationships between climatic parameters with dislodgeable 2,4-D residues following an application on a simulated soccer field.^a, b^

	^_____________________________________^ % 2,4-D dislodged of applied ^_____________________________________^			
Climatic parameter	1 DAT	2 DAT	3 DAT	6 DAT
	^______________________________________________________^ *r* ^______________________________________________________^			
RH	0.69^†^^c^	0.62^†^	0.57**	0.41
(AT—DP)	-0.73^†^	-0.62***	-0.55**	0.39
TFS	-0.82^†^	-0.69^†^	-0.58***	-0.45*
LW	0.58**	0.54**	0.23	0.53**

^a^ Abbreviations: DAT, d after treatment; RH, relative humidity; AT, air temperature; DP, dew point; TFS, time from sunrise; LW, leaf wetness.

^b^ Climatic conditions recorded on site at the Lake Wheeler Turfgrass Field Lab (Raleigh, NC, USA).

^c †^, ***, ** and * denote significance at P < 0.0001, 0.001, 0.01 and 0.05, respectively.

## Discussion

Data from this research align with previous reports that irrigation following foliar pesticide applications to turfgrass significantly reduce dislodgeable residues [24, 26, 27, 48]. Thompson et al. [28] reported dislodgeable 2,4-D declined to < 0.01% of the applied after a rainfall event 1 h after treatment. The less pronounced effect of water inputs in the presented research is likely due to a longer period of time between 2,4-D application and irrigation/rainfall (23 h). Although it cannot be determined from our data how 2,4-D was proportionally reduced in or on turfgrass vegetation following irrigation, this management practice significantly reduced the total 2,4-D in/on turfgrass vegetation at all sample timings. Averaged over experimental runs, irrigation reduced the total load of 2,4-D in/on turfgrass vegetation by 56% 2 DAT. This agrees with a previous report that 50% of applied dicamba, a synthetic auxin herbicide with physicochemical properties similar to 2,4-D, was lost via washoff after an 8 mm rainfall event [49].

Across irrigation treatments and sample collection timings, few differences were detected when reporting 2,4-D dislodgement as a percent of the total load in/on turfgrass vegetation. This can be misleading from a human exposure perspective because of the reference point that dislodgeable 2,4-D was calculated from, as there were differences between the total 2,4-D in/on turfgrass vegetation. This suggests that over time there is a general proportion of the total 2,4-D load in/on turfgrass vegetation that was dislodgeable via soccer ball roll and ultimately, management practices should be implemented to reduce these loads to limit human exposure.

Data from the TWD experiment suggested 2,4-D dislodgeability declined from morning to afternoon, which is hypothesized to be in part from turfgrass canopy moisture development. Poor to moderate correlations between dislodgeability and leaf wetness is attributed in part to sub-optimization of sensor placement (0.6 m height) in relation to the turfgrass canopy (0.03 m height). Kruit et al. [50] reported grass leaf wetness detection accuracy improved nearly 2-fold when sensors were moved from 1 to 0.1 m. Furthermore, previous research has shown turfgrass leaf wetness is commonly underestimated with data collected via flat-plate leaf wetness sensors [51, 52]. When using the RH threshold (71%) for grass moisture development determined by Kruit et al. [50], RH recordings in the presented research support 2,4-D dislodgeability is affected by canopy moisture. While this threshold will likely vary based on site-specific conditions, the strong positive correlations between RH and 2,4-D dislodgeability at 1 and 2 DAT in the presented research support this climatic parameter is an influencing factor. Additionally, 2,4-D dislodgement increased as AT approached DP and TFS decreased. Although it was not directly measured in this research, the aforementioned climatic parameters are predominately associated with dew formation on the turfgrass canopy. Once dew forms on turfgrass, 2,4-D may re-suspend on turfgrass vegetation, making it more readily dislodged. In addition to re-suspension, previous research has detected pesticides in plant guttation [53, 54]. Due to 2,4-D’s acropetal movement in plants, guttation may contribute to increased dislodgement detected at 5:00, 7:00 and 9:00 [14].

Previously published pesticide dislodgment research in turfgrass has not emphasized the TWD that sample collection occurs. Reports either do not state specifically when samples were collected, or they were collected at times later than 10:50 [21, 24, 26–28, 55]. Furthermore, risk assessments for human pesticide exposure from treated turfgrass make calculations based on the total h per d an individual is exposed to a treated area [56, 57]. For example, the only study specifically evaluating human 2,4-D exposure from treated turfgrass in the 2005 US Environmental Protection Agency’s (EPA) re-registration of 2,4-D was conducted at 12:30:00 (1 and 24 h after application) [8, 25]. Data from our research suggest 2,4-D risk assessments may be improved if more specificity is provided when calculating exposure potential by including atmospheric and turfgrass canopy conditions.

To better communicate potential human 2,4-D exposure from this research, 2,4-D exposure d^-1^ was calculated with the algorithm obtained from the 2012 EPA Standard Operating Procedures for Residential Pesticide Exposure (Post-Application dermal Exposure—Physical Activities on Turf):

$${\text{E = TTR}}_{\text{t}}\text{ x CF1 x TC x ET}$$ (3)

where E = exposure (mg d^-1^); TTR_t_ = turf transferable residue on day t (0.82 μg cm^2^); CF1 = unit conversion factor (0.001 mg μg^-1^); TC = transfer coefficient (49,000 cm^2^ hr^-1^; 1 to 2 yr children); and ET = exposure time (1.5 hr d^-1^; 1 to 2 yr children) in the algorithm [57]. The algorithm, TC and ET were obtained from the EPA risk assessment, while TTR_1_ (0.82 μg 2,4-D cm^2^) corresponds to the maximum amount dislodged in the presented research (1 DAT– 5:00) [57]. It was calculated that a human would be exposed to 60 mg 2,4-D d^-1^, which is adjusted to 6 mg after a 10% 2,4-D dermal absorption rate [8, 57]. Using the EPA risk assessment value for short-term (30 d) human dermal exposure of 25 mg kg^-1^ d^-1^ it, was determined that an average 1 to 2 yr child (11 kg) may be dermally exposed to 275 mg 2,4-D d^-1^ without adverse effect [8]. By calculating our observed maximum daily exposure (6 mg 2,4-D d^-1^) as a percent of the short-term daily dermal exposure allowance without adverse effect (275 mg 2,4-D d^-1^), it was determined that 2,4-D dislodged from one ball roll over a 0.45 m^2^ area equaled 2.2% of this limit. While the application rate in the presented research was 20% greater than current label allowances on athletic fields, which overestimates real-world exposure with this algorithm, the area covered by one ball roll in this research equals 0.18% of the area of the smallest children’s (U6) soccer field (14 by 18 m) recommended by the US Youth Soccer Organization [39].

## Conclusions

This research evaluated 2,4-D dislodgement from hybrid bermudagrass, the most common athletic field turfgrass in tropical and subtropical regions, with a method simulating a common process in soccer, the most popular international sport. Our findings indicate that 2,4-D residues can dislodge from hybrid bermudagrass up to 7 DAT. Ultimately, 2,4-D’s very high K_s_ coupled with low K_oc_ have a substantial impact on dislodgeability, as irrigation reduced dislodgement > 68% and conditions favoring turfgrass canopy dryness reduced dislodgement to ≤ 0.1% of the applied from 2 DAT until the end of the study, regardless of irrigation.

In conclusion, human 2,4-D exposure on hybrid bermudagrass athletic fields may be minimized by the coordination of pesticide applications with event scheduling. By coupling the effect of irrigation after an application with dislodgeability decreasing when the turfgrass canopy is dry, data suggest 2,4-D may not be dislodgeable in the afternoon at, or beyond 2 DAT. However, without further research to investigate this observation, workers and non-workers should enter bermudagrass athletic fields recently treated with 2,4-D with caution in the days following an application. Future research should investigate similar research objectives with irrigation applied in the morning when 2,4-D is most dislodgeable, evaluate dislodgeability of additional pesticides from alternative turfgrass species, as well as the effect of adjusting application practices such as spray nozzle or carrier volume to reduce dislodgeable pesticide residues from turfgrass.
